# Decoding the Effect of Running on Flavor Perception Changes during Consumption of Sports Drinks

**DOI:** 10.3390/foods13081266

**Published:** 2024-04-20

**Authors:** Biwen Pu, Ruixin Meng, Yige Shi, Dandan Pu

**Affiliations:** 1 Department of Physical Education and Sport Research, Guangdong University of Finance, Guangzhou 510521, China; 18-071@gduf.edu.cn; 2 School of Physical Education and Sport Science, South China Normal University, Guangzhou 510006, China; 3 China Key Laboratory of Geriatric Nutrition and Health, Ministry of Education, Beijing Technology and Business University, Beijing 100048, China; mengrx0701@163.com (R.M.); jennyshiyige@163.com (Y.S.); 4 Key Laboratory of Flavor Science of China General Chamber of Commerce, Beijing Technology and Business University, Beijing 100048, China; 5 Food Laboratory of Zhongyuan, Beijing Technology and Business University, Beijing 100048, China

**Keywords:** sports drink, flavor compounds, temporal dominance of sensations, gas chromatography-mass spectrometry, recognition threshold

## Abstract

An online survey was conducted to show that most respondents preferred sports drinks with sweet and fruity characteristics. Eleven sports drinks with higher consumers’ preferences were further selected for aroma and taste evaluation. Temporal dominance of sensations analysis showed that fruity and fresh attributes were dominant, while sour and fruity sweet were dominant tastes during consumption. β-Damascenone, β-ionone, and linalool contributing to floral perception, γ-decalactone, ethyl cinnamate, and isoamyl acetate contributing to fruity perception, and menthol contributing to fresh perception were confirmed by odor activity value analysis. Running affected the nasal air flow and the saliva secretion, resulting in the flavor perception changing from fruity sweet, sweet, and fruity to sour because the recognition threshold decreased for sweet, fruity, floral, and fresh flavors and increased for saltiness, astringency, and sour tastes.

## 1. Introduction

Sports drink consumption is not only popular among athletes but also attracts increasing attention from ordinary consumers. Sports drinks are one of the four functional beverages (sports drinks, energy drinks, nutrient drinks, and the others), and the market size of sports drinks in China is expected to reach 21.582 billion yuan in 2024 [1]. “Health and function” is becoming a new trend in the Chinese beverage market [2,3]. The beverage industry is currently booming. Moreover, consumer demand for nutrient-fortified beverages and health, tea, and plant protein drinks is increasing. Sports drinks can help resist fatigue and supplement energy, and they are increasingly favored by consumers [4]. Among them, sports drinks account for >50% of the market share of functional drinks. Therefore, research on the aroma and function of sports drinks is becoming an important direction.

Currently, fruity is the most popular flavor both domestically and internationally [5], and the popular fruity flavor themes are constantly updated. The citrus, grapefruit, and herbaceous plant (elderberry) flavors have been the most popular in recent years. In addition, driven by healthy diet trends, consumers hope to improve their immunity by consuming healthy food and functional beverages. Citrus fruits have attracted strong demand from these consumers. Moreover, plant-based flavors, such as elderberry, not only enhance immunity and resist influenza viruses but also alleviate anxiety, promote sleep, and are popularly accepted by consumers [6].

Sports drinks, which comprise water, sugar, electrolytes, and nutrients with refreshing or antifatigue effects, can quickly replenish the water, electrolytes, and sugars lost during exercise [7]. Although many types of sports drinks are commercially available with variable functional characteristics, flavor quality remains the key factor determining consumer preference. A pleasant sensory experience not only allows athletes to enjoy the drink but also alleviates their tension and relaxes them psychologically, which is conducive to the active development of competitive activities [8,9]. However, the flavor perception varies under different sport states due to changes in retronasal air flow and salivary secretion [10,11]. Therefore, the flavor preferences of athletes based on actual sport scenarios, changes in flavor perception during the exercise process, and key factors affecting flavor perception changes should be investigated and clarified. Thus, characterization of the flavor perception changes during exercise will help develop sports drinks with distinct flavors that meet the preferences of sport populations. However, few studies have assessed the flavor changes of sports drinks during exercise.

Currently, dynamic sensory evaluation, including dynamic quantitative descriptive analysis [12], temporal dominance of sensations (TDS) [13,14], and temporal check-all-that-apply [15], are the most popular analytical methods applied in flavor perception during oral processing or at different times that can intuitively reflect consumer real-time perception of food flavor [16]. These methods can also help distinguish the subtle differences among variance samples. Therefore, the aims of this study were to: (1) investigate the consumer preference and recognition of sports drinks and screen the sports drinks with higher consumer preference; (2) evaluate the flavor changes while drinking the sports drink before and after running by the TDS method; (3) determine the changes in physiological parameters, including the exhalation volume through the nasal cavity and saliva secretion after running; and (4) characterize the flavor recognition threshold value changes before and after running.

## 2. Materials and Methods

### 2.1. Materials and Chemicals

Twenty-two sports drinks based on the online survey results were purchased from the local supermarket (Beijing, China) with the latest launch time (June 2023). The detailed information for ten different brands of sports drinks are presented in Table 1. Food-grade sodium glutamate (99.9%) was purchased from Shenyang Hongmei Enterprise Group Co., Ltd. (Shenyang, China). Food-grade citric acid (99.9%) was purchased from Zhengzhou Zhongsi Food Co., Ltd. (Zhengzhou, China). Drug-grade quinine (99.8%) was produced by Hubei Shixing Chemical Co., Ltd. (Wuhan, China). Food-grade fructose syrup (99.5%) was purchased from Wenzhou Heji Food Co., Ltd. (Wenzhou, China). Food-grade menthol (99.9%), ethyl cinnamate (99.9%), and linalool (99.9%) were purchased from Firmenich (Geneva, Switzerland). Food-grade fructose syrup (99.9%) and sucrose (99.9%) were bought from Huai’an Ruixiang Food Factory (Huai’an, China). Food-grade citric acid (99.9%) was produced by Jiangsu Ruiduo Biotechnology Co., Ltd. (Xuzhou, China). Food-grade tea polyphenols (98%) were produced by Xi’an Lishi Biotechnology Co., Ltd. (Xi’an, China). Food-grade sodium chloride (99.1%) was purchased from Sichuan Jiuda Penglai Salinization Co., Ltd. (Suining, China). 2-Methyl-3-heptanone and methanol (99.9%) were purchased from J&K (Beijing, China).

### 2.2. Online Survey

A total of nine questionnaires were set in the online survey, including the gender, age, province of residence, occupation type, frequency of regular exercise, do you know about sports drinks, which types of sports drinks do you know about, which flavor characteristics of sports drinks you like, and why flavor is important for athlete drinks.

### 2.3. Sensory Evaluation

Seventeen healthy, nonsmoking panelists (9 females and 8 males, aged 24–30) who did not have rhinitis were recruited from our laboratory. All participants were informed of and agreed to conduct the sensory valuation tests and the physiological parameter measurements during running, and all participants received monetary rewards. The experiment was approved by the Ethics Committee of Beijing Technology and Business University (BTBU202333). The ranking test was conducted by asking the panelists to sort the sports drink samples according to their preferences.

Flavor perception training. Taste perception training was conducted by TDS according to our previous work [14]. Five basic taste solutions with varied concentrations were presented to the panelists, who were asked to rank these different taste solutions according to their perception intensity. For the aroma perception training, panelists were asked to smell 54 types of aroma (Le Nez duVin ^®^) and were requested to distinguish their differences. Flavor perception training lasted for 3 weeks (twice a week, with each training lasting for 1 h) before TDS analysis.

Flavor description collection. Each panelist was requested to record the aroma and taste descriptions during sports drink consumption. A total of 25 flavor attributes, including sour, bitter, sweet, fruity sweet, saltiness, umami, astringent, metallic, earthy, fruity, green, spicy, woody, floral, caramel-like, mango, pineapple, blueberry, peach, fresh, lemon, grapefruit, orange, tasteless, and odorless, were determined according to their higher frequency. Finally, eight attributes, including sour, sweetness, fruity sweetness, bitter, saltiness, fruity, floral, and fresh, were determined.

TDS analysis. The TDS method can identify the dominant flavor attributes of sports drinks during drinking and accurately describe their variation along with the oral processing time. During TDS analysis, panelists were required to identify only the dominant aroma/taste characteristics from the final eight attributes without determining the intensity [17]. A 30 mL volume of sports drink was poured into the oral cavity. Eleven oral processing time blocks (0, 10, 20, 40, 60, 80, 100, 120, 140, 160, and 180 s) were used. Panelists were requested to select one dominance attribute from the eight flavor profiles. The TDS curves were generated as previously described [8,18]. The changes in flavor perception at different running times (0, 5, 10, 15, 20, and 25 min) were evaluated by TDS while drinking the sports drink. Panelists had a 10-min rest after each running time block.

### 2.4. Headspace Solid-Phase Microextraction (SPME)

To characterize the aroma compounds contributing to aroma perception during sports drink consumption, the volatile compounds in sports drinks were extracted via SPME. The volatile compounds in sports drink samples were extracted with an automatic headspace sampling system (TriPlus RSH, Reinach, Switzerland) with a 65 μm fiber (1 cm length of fiber, polydimethylsiloxane/divinylbenzene, Supelco, Bellefonte, PA, USA). Sports drink samples (4 mL) were loaded into the SPME vial (20 mL), and 1.20 g of NaCl was added. A 5 μL volume of internal standard (2-methyl-3-heptanone, 0.10 mg/mL) was dissolved in methanol. Samples were incubated for 20 min at 38 °C (a simulation of the temperature of the oral cavity). Subsequently, the SPME fiber was inserted into the headspace of the extraction bottle to adsorb the volatile compounds for 40 min at 38 °C. After extraction, the loaded SPME fiber was immediately injected into the injection port of the gas chromatography-mass spectrometry (GC-MS) to desorb for 5 min at 250 °C. Volatile compounds were quantified (semiquantitative analysis) by dividing the peak areas of the compounds of interest by the peak area of the internal standard. All samples were repeated in triplicate.

### 2.5. Gas Chromatography-Mass Spectrometry (GC-MS)

The Agilent 8890 gas chromatography equipped with 5977B mass spectrometry was used in this study. Samples were analyzed on DB-WAX fused-silica capillary columns (30 m × 0.25 mm i.d., 0.25 μm film thickness, Agilent, Santa Clara, CA, USA). Helium (99.999%) was used as the carrier gas, and the column flow rate was 1.00 mL/min in pulsed splitless injection mode. The injector temperature was 250 °C. The initial oven temperature was 35 °C, which was held for 1 min. The temperature was then increased to 100 °C at 4 °C/min (held for 1 min), increased to 170 °C at 2 °C/min (held for 1 min), increased to 220 °C at 5 °C/min, and finally held for 1 min. The mass spectral detection conditions were: mass detector temperature of 150 °C; electron impact mode of 70 eV; ion source temperature of 240 °C; transmission line temperature of 250 °C; and mass ranges from *m*/*z* 40 to *m*/*z* 450 in full scan mode.

### 2.6. Physiological Parameters with Different Running Times

The airflow in the retronasal cavity has a considerable effect on aroma perception [8,10]. Since directly monitoring the retronasal exhalation flow volume of panelists is difficult during exercise, an equivalent measurement was achieved by collecting the total volume of a single retronasal exhalation. The single nasal exhalation method was conducted to collect the air volume in a disposable air collection bag. Five repeated tests were performed at each running time block. Sixteen volunteers with daily exercise habits were selected from the sensory evaluation panelists described in Section 2.3, Sensory Evaluation. The exercise method was running at different times (0, 1, 5, 10, 15, 20, and 25 min). The collected air bag was measured using a JCH-2400 intelligent dual constant current atmospheric sampler (Qingdao Juchuang Environmental Protection Group Co., Ltd., Qingdao, China). The saliva secretion affected the delivery of the taste compounds in the mouth cavity. The salivary secretion was collected by adding a medical cotton ball (0.45 g) into the mouth cavity at different time points to adsorb saliva. The saliva secretion amount was determined by the weight difference of the medical cotton ball before and after running. Each test was repeated in triplicate.

### 2.7. Perception Threshold Value Changes of the Flavor

The physiological parameters of the human oral cavity and the regulation of the body undergo significant changes after movement, causing changes to flavor perception. Key aroma compounds contributing to the sports drink aroma perception were screened according to the aroma identification and odor activity value calculation results. The dominant taste attributes (determined from the TDS results) were selected, and the corresponding taste contributors were confirmed from the main taste ingredients of the sports drinks. Therefore, 9.00 g/L fructose syrup (fruity sweetness), 7.00 g/L sucrose (sweet), 2.00 g/L citric acid (sour), 2.00 g/L tea polyphenols (astringent), and 6.00 g/L sodium chloride (saltiness) were used to study changes in taste perception thresholds before and after exercise. A series of 10 2-fold dilutions of the flavor compounds were presented to the panelists before and after running for 5 min. Solution samples (two water solutions and one aroma/taste solution) coded with three-digit numbers were poured into the mouth cavity, and panelists were requested to select the different sample based on the recognition of the aroma or taste quality. The final recognition threshold was determined through statistical evaluation of the perception of aroma and taste by the evaluators during the tasting process, and the difference between the recognition threshold and that after 5 min of exercise was compared.

### 2.8. Statistical Analysis

Data were analyzed via one-way analysis of variance (Duncan test) using SPSS 22.0 (SPSS Inc., Chicago, IL, USA). The dominance rates (%) of each attribute were calculated and plotted against the chewing time using Microsoft Excel 2016. The TDS curves were performed by Origin Pro 9.1 (OriginLab Corporation, Northampton, MA, USA).

## 3. Results

### 3.1. Respondent Recognition of Sports Drinks

**Figure 1:**
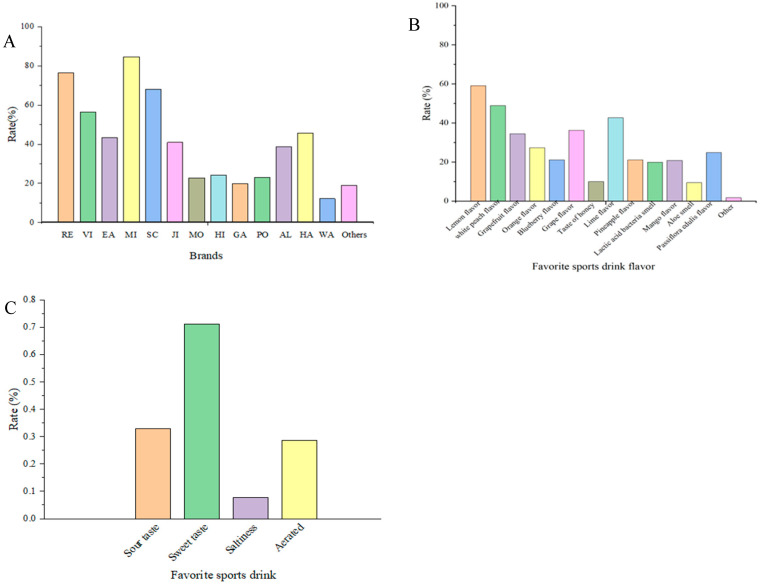
The online survey results for sports drinks ((**A**) consumer’s favorite brands of sports drink; (**B**) consumer’s favorite characteristics of sports drinks; (**C**) consumer’s favorite taste of sports drink).

A questionnaire survey (403 consumers) was conducted on 22 brands of common sports drinks from the market. The survey results showed that MI, RE, SC, VI, HA, EA, JI, AL, HI, PO, MO, and EA were the top 12 brands of sports drinks (Figure 1A). Aroma preference results showed that consumers preferred fruity aromas of lemon, white peach, lime, grape, grapefruit, orange, passiflora edulis, blueberry, pineapple, and mango attributes (Figure 1B). Flavor quality determines the psychological pleasure of consumers, which plays a decisive role in consumer preferences. Currently, sweet and fruity flavor attributes are the most popular types of sports drinks (Figure 1C). The results of the online survey elucidated that the flavor was important to improve the consumer’s preference. Therefore, the flavor quality of sports drinks should be investigated to help consumers understand the flavor characteristics and perceptions of sports drinks.

### 3.2. Panelists’ Preference Test

According to the recognition and preference results from the online survey (Figure 1A), 22 brands of sports drinks were selected (Table 1) for ranking test. Sports drinks were divided into two groups for ranking analysis due to the number of samples and similar flavor characteristics. Subsequently, the sports drink samples with unpleasant attributes, including metallic, bitter, astringent, and earthy attributes, were discarded. Samples with lower overall flavor intensity were also discarded for further sensory evaluation. Finally, eleven sports drink samples, including MI (pineapple, peach, orange, and green lemon flavors), HI-classic flavor, HA (lemon flavor), SC (grapefruit flavor), GA (blueberry flavor), and VI (lemon, blueberry, and orange flavors), were associated with high panelist preference (high ranking). Based on the analysis of the flavor attribute frequency, the main flavor profiles of these eleven sports drinks were determined, including sweet, sour, saltiness, fruit sweetness, floral, fruity, and fresh. The sweet flavor referred to the sucrose taste, while the fruit sweetness corresponded to the taste of fructose syrup.

### 3.3. TDS Analysis

The TDS curves of eleven sports drinks during consumption are shown in Figure S1. Most sports drinks had more than four flavor attributes over the significance line, and the fruity, fresh, fruity sweet, and sour perceptions were the dominant attributes. Based on the dynamic flavor attribute perception during oral processing, two classes could be obtained: (1) dominated by the fruity, fruit sweet, and fresh attributes (MI-pineapple, MI-peach, and HA-lemon flavors), and (2) dominated by the sour, fresh, and fruit sweet attributes (the remaining eight brands of sports drinks). The dominant rates of fruity, fresh, sour, and fruit sweetness attributes were above the significance line during oral processing, indicating these four flavor attributes were the dominant characteristics of these sports drinks. The floral attribute above the significance line in MI-orange, HI-classic, and VI-orange flavor sports drinks. Interestingly, the saltiness and astringent attributes were lower than the chance line, indicating that these two flavor attributes did not significantly contribute to the flavor perception of sports drinks. According to the duration time of the flavor perception, the MI-pineapple flavor (fruit sweet, fresh, fruity, and floral), MI-peach flavor (sour, fruity, and fresh), HI-classic flavor (saltiness, sour, and fruity), and VI-blueberry flavor (saltiness, sour, and fruit sweet) had more than three attributes with a retention time > 100 s. Based on the alternating change patterns of flavor attributes, the perception of six sports drink samples was a sour perception because of the highest dominance rate while that of three other drinks was mainly the fresh attribute. The remaining two sports drinks were mainly perceived for their fruity and fruity sweetness flavors.

### 3.4. Principal Component Analysis (PCA) Analysis

**Figure 2:**
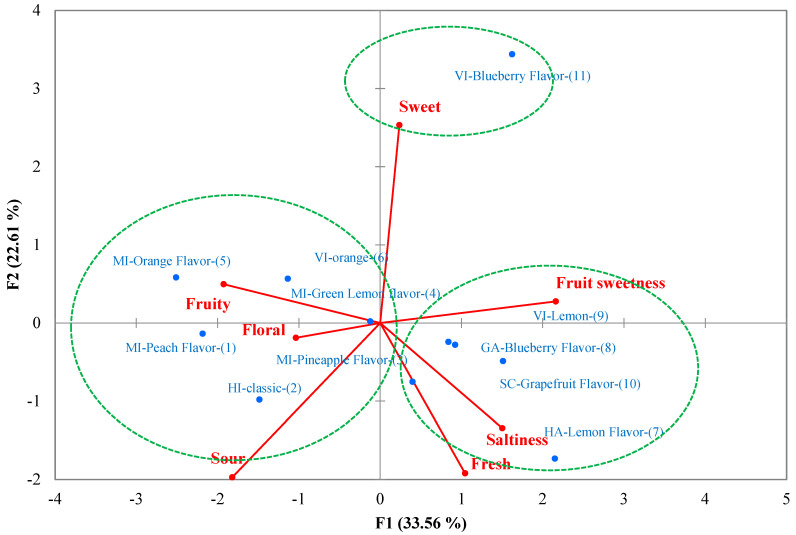
Principle component analysis results of the duration time of sports drinks during oral processing (the numbers behind each of the sports drinks represent the consumers’ preference rank number).

PCA was conducted based on the duration of different sensory attributes (Figure 2). The 11 samples could be divided into three categories: (1) MI-orange, MI-lime, MI-peach, VI-orange, and HI-classic flavors were located at the negative axis of the first principal components with strong fruity, floral, and sour perception; (2) MI-pineapple, VI-lemon, SC-grapefruit, and HA-lemon flavors were located at the positive axis of the first principal component and the negative axis of the second principal component with strong fruit sweetness, saltiness, and fresh perception; (3) VI-blueberry flavor was located at the positive axis of the first and second principle components with a strong sweet attribute. In addition, the ranking of panelist consumer preferences was consistent with the cluster analysis results; the first class had the highest ranking of panelist preference from 1 to 6, whereas the second category was ranked mainly from 7 to 9, and the final category was ranked last. The cluster and ranking test results confirmed that the higher preference sports drinks had more complex flavor change patterns during oral processing. The PCA analysis was consistent with the TDS analysis, where fruity, floral, and sour perceptions were the key characteristics of sports drinks that determined consumers’ preferences.

### 3.5. Changes in Oral Parameters with Running Times

Nasal airflow is the main channel transmitting aroma compounds to the olfactory epithelial cells, and sniffing is therefore a common behavior that enhances aroma perception [19]. The changes in nasal airflow affect the aroma concentration received by olfactory epithelial cells, resulting in variances in the olfactory perception signal by nerves associated with the olfactory system. At the resting state, approximately 10% of the inhaled air reaches the olfactory epithelium area, and the nostrils can quickly draw gas from the environment in a turbulent pattern, causing more aroma molecules to be deposited on the olfactory mucosa to promote aroma perception [10,20]. Oral processing is a complex dynamic process, and the transportation of aroma compounds to the retronasal nasal cavity is also a series of alternating dynamic events. Therefore, monitoring the airflow changes in real-time is key to analyzing the aroma transportation in the retronasal nasal cavity.

**Figure 3:**
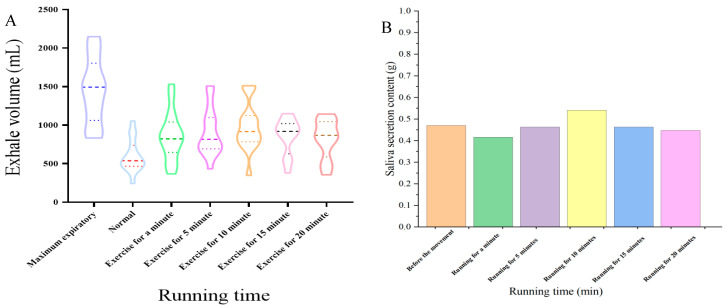
Oral parameter changes during running ((**A**) exhale volume of single exhalation through the nasal cavity; (**B**) saliva secretion with different running times).

The maximum volume of exhalation through the nasal cavity for a single exhalation experiment is shown in Figure 3A. The maximum volume for a single exhalation through the nasal cavity was 1282 mL, but the normal average volume of a single exhalation through the nasal cavity at resting state was 536 mL. After 1 min of running, the nasal-exhaled volume significantly increased and continued to gradually increase with the extension of running time. After 5 min of exercise, the single nasal-exhaled volume was 870 mL, and at 10 min, this reached the peak value (940 mL). After 15 min of running exercise, the single nasal exhalation volume decreased to 870 mL and then to 860 mL after 20 min of running exercise, with no significant difference (*p* > 0.05). These volume changes of a single nasal exhalation suggested that the nasal flow rate rapidly increased with running time within 10 min, stabilized at 15 min, and decreased after 20 min of exercise. The nasal air flow could therefore be divided into three stages: rapid increase (0–10 min), stable (10–15 min), and decrease (≥15 min).

The oral saliva secretion at different exercise times is shown in Figure 3B. The amount of saliva secretion first increased and then decreased, with the highest saliva secretion reaching 0.55 g at 10 min of exercise. After 15 min of running, saliva secretion rapidly decreased to 0.48 g, with subtle changes from 15 to 20 min. Therefore, considering the nasal airflow volume and saliva secretion change patterns during running, flavor changes during sports drink consumption affected by exercise were investigated from 0 min (resting state) to running for 10 min.

### 3.6. Dynamic Flavor Perception of Sports Drinks with Different Running Times

**Figure 4:**
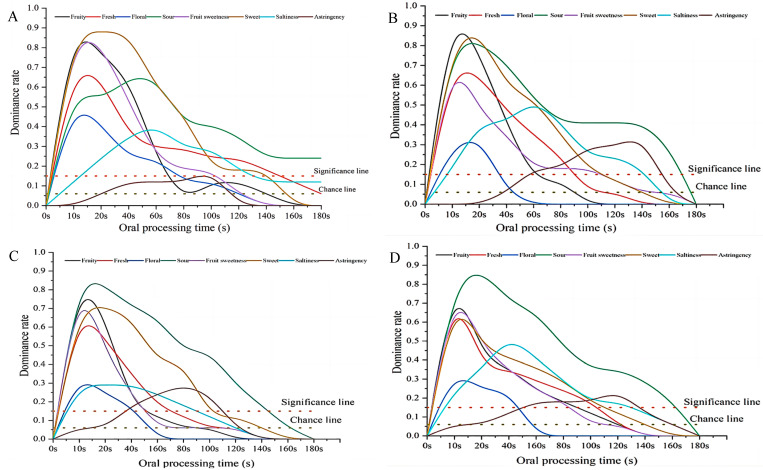
Dynamic flavor perception during drinking of MI-peach flavor with different running times ((**A**), running 0 min; (**B**), running 1 min; (**C**), running 5 min; (**D**), running 10 min).

The dynamic flavor perception during sports drink oral processing at different running times was performed, and four change patterns were observed (Figure 4): (1) Compared with that at the resting state (Figure 4A), the sweet and fruit sweetness flavors decreased along with running. (2) The dominant rate of sour perception increased along with running and became the dominant note after 5 min (Figure 4C). (3) The dominant rates of fresh and floral perception decreased after running and stabilized at the dominant rates of 0.6 and 0.3, respectively. The duration of floral perception decreased from 140 to 70 s, and with fresh perception, from 180 to 140 s. (4) The fruity, salty, and astringent perception increased within 5 min and then decreased after 10 min of running. Notably, the astringent attribute could not be perceived in the resting state but was significantly perceived after running. Changes in aroma perception patterns (fresh, floral, and fruity) indicated that the aroma perception increased along with a certain increase in nasal flow volume because of running, although transportation concentrations can decrease when nasal flow volume increases [12,19]. The duration of sweet and fruity sweet flavors were almost the same, but the dominant rate decreased from 0.85 to 0.60.

Overall, running could increase the nasal air flow volume, resulting in changes in flavor perception. Running could also affect saliva secretion to alter the transportation of taste compounds during oral processing [21]. After running, the dominant perception during consumption of the sports drink changed from fruity sweet, sweet, fruity to fruity, and sour within 5 min. However, after 10 min of running, the dominant flavor perception subsequently changed to sour, the perception of which was significantly higher than that for the other attributes.

### 3.7. Identification of Aroma Compounds in Sports Drinks

A total of 45 aroma compounds, including 13 alcohols, 11 esters, 6 aldehydes, 5 lactones, 4 ketones, 3 olefins, 2 acids, and 1 thiazole, were identified in the MI-peach flavor sports drink at 38 °C (Table 2). The relative content results showed that γ-decalactone with peach-like and coconut-like notes had the highest content (1458.77 μg/L), followed by linalool (1331.53 μg/L) with floral and sweet attributes, terpineol (1106.38 μg/L) with pine, terpene, and green notes, benzaldehyde (870.21 μg/L) with bitter almond-like and green attributes, and γ-undecalactone (660.65 μg/L) with peach-like and sweet notes. The concentration of the remaining aromatic compounds was <500 μg/L.

To investigate the aroma contribution to the overall aroma profiles, their odor activity values (OAVs) were calculated based on the concentration compared to the threshold values in the water matrix. A total of 39 aroma compounds had an OAV ≥ 1, and nine compounds had an OAV > 1300. Among them, β-damascenone had the highest OAV of 164,266, followed by β-ionone (39,243), terpineol (7376), γ-octanoic lactone (3957), benzaldehyde (2486), citronellol (1881), γ-decalactone (1326), and linalool (1530). Fifteen compounds (1, 3, 8–10, 15, 17, 21, 25–26, 34, 43–46) had an OAV from 104 to 835. Higher OAV values indicated that the compounds play a key role in fthe lavor of MI-peach flavor sports drink. Therefore, according to the aroma quality and OAV values, β-damascenone, β-ionone, and linalool contributed to floral perception, γ-decalactone, ethyl cinnamate, isoamyl acetate, and hexyl acetate contributed to fruity perception, and menthol contributed to fresh perception and were selected for further analysis.

### 3.8. Changes in Flavor Thresholds before and after Running

**Figure 5:**
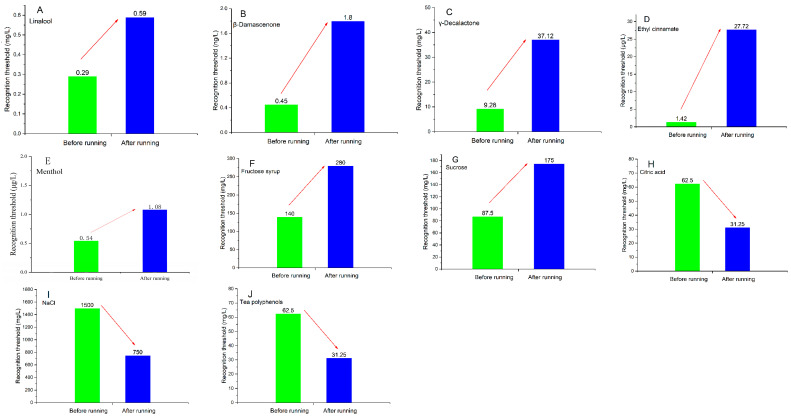
Recognition threshold change results of different flavor compounds before and after running ((**A**), linalool; (**B**), *β*-damascenone; (**C**), *γ*-decalactone; (**D**), ethyl cinnamate; (**E**), menthol; (**F**), fructose syrup; (**G**), sucrose; (**H**), citric acid; (**I**), NaCl; (**J**), tea polyphenols).

To investigate the changes in flavor perception of sports drinks during consumption at different running times, changes in the recognition threshold of different flavor compounds before and after running were detected. The TDS results, aroma identification, and OAV analysis confirmed that menthol contributed to fresh perception, ethyl cinnamate, *β*-damascenone, and *γ*-decalactone contributed to fruity aroma, and linalool contributed to the floral aroma. The recognition threshold (RT) value change results are shown in Figure 5.

After running, the RT of saltiness decreased from 1500.00 to 750.00 mg/L (Figure 5I), indicating an increased sensitivity to saltiness. This may be due to the body excreting more salt and increasing demand for salt intake to balance the signal regulation of saltiness perception [25]. The RT of sour taste also decreased. The RT of citric acid decreased from 62.50 to 31.25 mg/L (Figure 5H), causing an increase in sour taste perception in sour drinks of the same concentration. This result explained why the sensibility of acidity perception increased after exercise [26]. The RT of fruit sweetness (fructose syrup) increased from 140.00 to 280.00 mg/L, and sweetness (sucrose) increased from 87.50 to 175.00 mg/L, indicating that after exercise, a higher intensity of sweetness stimulation was needed to stimulate the taste buds. Therefore, the sweetness perception of beverages after exercise significantly decreased, which also confirmed that the body needed to replenish energy during movement. However, a previous study reported that the sweet perception (100–200 mM) was not changed during hiking [7]. Phenolic compounds combine with tyrosine-rich proteins in saliva to form precipitates, which are the main mechanism for producing astringency [26]. The RT of astringency decreased from 62.50 to 31.25 mg/L after exercise. With the extension of exercise time (0–10 min), saliva secretion showed an increasing trend in the content of saliva protein, resulting in more protein binding to phenolic compounds and the perception of strong astringency [27].

The RT of menthol increased from 0.54 to 1.08 mg/L (Figure 5E), although the duration of mint perception in the oral cavity was shorter. Therefore, the fresh perception in the oral cavity slightly weakened along with the extension of exercise time. After exercise, the RT of linalool (floral aroma) increased from 0.29 to 0.59 mg/L, that of cinnamate (fruit aroma) increased from 1.42 to 27.72 µg/L, and that of γ-decalactone (fruity aroma) increased from 9.28 to 37.12 µg/L. Thus, the RT of aroma compounds in oral processing increased along with running. Since the respiratory airflow increased, their transfer to the posterior nasal cavity decreased. The RT value of β-damascenone (contributing to both floral and fruity notes) in the mouth cavity was 0.45 ng/L but increased to 1.80 ng/L after running for 5 min. Therefore, the perception intensity of fresh, fruity, and floral attributes gradually weakened along with the increasing exercise time.

## 4. Conclusions

An online survey showed that good flavor quality and functionality were the two main driving forces behind respondents preference for sports drinks. Of the respondents, 34.6% believed that drinking sports drinks could restore physical strength, alleviate fatigue, and be refreshing. However, respondents were less familiar with the brands and functional properties. TDS and PCA analysis elucidated that 11 types of sports drinks with high respondent preference were divided into four categories, of which fruity, floral, and sour were the dominant flavor attributes. Dynamic flavor perception analysis elucidated that the perception of sweet, fruit sweetness, floral, and fresh attributes decreased along with running but that of sour, salty, and astringent attributes increased after running. Odor activity value analysis confirmed that β-damascenone, β-ionone, and linalool contributed to the perception of floral aromas, γ-decalactone, ethyl cinnamate, and isoamyl acetate contributed to fruity aromas, and menthol contributing to fresh aromas. Changes in the RT value confirmed that the dominance rate of aroma perception decreased because of the increase in the RT of the corresponding aroma compounds due to changes in retronasal air flow after running. The dominance rate of astringent, salty, and sour perceptions increased because of the decrease in their RT values in corresponding taste compounds that resulted from the secretion of saliva after running.

## Figures and Tables

**Table 1:** Detailed information on ten brands of sports drinks.

Brands	Flavor Characteristics ^#^	Manufacturer
MI **	Pineapple flavor (1), peach flavor (2), orange flavor (3), lime flavor (4)	Danone (China) Food and Beverage Co., Ltd. (Zhongshan, China)
HI *	Classic flavor (5)	Hebei Dali Food Co., Ltd. (Quanzhou, China)
MO *	Taurine flavor (6), Coca Cola flavor (7), Mango (8)	Jiangsu Taigu Coca Cola Beverage Co., Ltd. (Nanjing, China)
HA **	Fruity (9)	Taizhou Tongshi Enterprise Co., Ltd. (Taizhou, China)
JI *	Classic flavor (10)	Guangdong Jianlibao Co., Ltd. (Foshan, China)
RE *	Classic flavor (11)	Tiansi Medical and Health Care Co., Ltd. (Tianjing, China)
SC *	Lemon flavor (12), peach flavor (13), grapefruit flavor (14)	Nongfu Spring (Jiande) Xinanjiang Drinking Water Co., Ltd. (Hangzhou, China)
GA *	Blueberry flavor (15), orange flavor (16), orange flavor (17)	Yangzhou Dingjin Food Co., Ltd. (Yangzhou, China)
AL *	Peach flavor (18), lime flavor (19)	Yuanqi Forest (Anhui) Beverage Co., Ltd. (Chuzhou, China)
VI *	Blueberry flavor (20), lemon flavor (21), orange flavor (22)	Nongfu Spring (Jiande) Xinanjiang Drinking Water Co., Ltd.

^#^, represented the serial number of twenty-two sports drinks; *, flavor from the synthetic flavoring essence and fruit pulp; **, flavor from the synthetic flavoring essence and fruit pulp.

**Table 2:** The aroma quantification results of Mi-peach flavor sports drink.

No.	Aroma Compounds	CAS	Aroma Quality	RI		Relative Content (μg/L)	Threshold Value (μg/L)	OAV
				Referenced	Calculated			
1	Ethyl acetate	141-78-6	Fruity, sweet, solvent-like	872	883	342.24 ± 21.11	3.3 ^b^	104
2	Ethyl butyrate	105-54-4	Fruity, sweet	1028	1035	31.47 ± 3.68	0.76 ^a^	41
3	Isoamyl acetate	123-92-2	Banana-like, fruity	1115	1123	162.74 ± 20.06	0.4 ^b^	407
4	Limonene	5989-27-5	Lemon, citrus, fresh, green	1143	1145	91.38 ± 9.23	34 ^c^	3
5	*β*-Myrcene	123-35-3	Geranium-like, carrot-like, hop-like	1155	1157	61.36 ± 3.26	1.2 ^a^	51
6	Heptanal	111-71-7	Green, citrus-like, fatty	1185	1191	21.41 ± 0.15	9.5 ^b^	2
7	*trans*-2-Hexenal	6728-26-3	Green, grass, leaf	1201	1215	12.06 ± 2.17	0.284 ^b^	42
8	Hexyl acetate	142-92-7	fruity, pear-like	1275	1272	252.72 ± 12.39	0.48 ^b^	527
9	Terpinolene	586-62-9	Pine, terpene, woody	1271	1281	33.03 ± 1.55	0.2 ^b^	165
10	3-Methylbutyl 3-methylbutanoate	659-70-1	Fruity, sweet	1287	1290	87.78 ± 7.19	0.64 ^b^	137
11	cis-3-Hexenyl acetate	3681-71-8	Green, banana-like	1316	1303	3.1 ± 0.39	0.2 ^b^	16
12	(*Z*)-Hex-2-enyl acetate	56,922-75-9	Sweet, fruity	1305	1329	47.34 ± 4.01	13 ^a^	4
13	2-Isopropyl-4-methylthiazole	15,679-13-7	Garlic, coffee, and tropical fruity	1339	1349	14.05 ± 1.32	-	-
14	1-Hexanol	111-27-3	Grassy, marzipan-like	1360	1365	29.05 ± 2.23	0.5 ^b^	58
15	(*Z*)-3-Hexen-1-ol	928-96-1	Lettuce-like, green, leaf	1377	1381	395.72 ± 54.55	3.9 ^a^	101
16	Nonanal	124-19-6	Green, soapy	1390	1390	19.68 ± 7.56	2.8 ^a^	7
17	(*E*)-2-Hexen-1-ol	928-95-0	Sweet, ethereal	1409	1410	26.07 ± 1.54	0.1 ^b^	261
18	Acetic acid	64-19-7	Sour	1424	1440	438.47 ± 36.07	99,000 ^a^	<1
19	Isomentone	491-07-6	Fresh, mint-like	1465	1458	20.7 ± 4.35	-	-
20	Furfural	98-01-1	sweet, cereal-like	1461	1453	112.94 ± 12.89	3 ^b^	38
21	Octyl acetate	112-14-1	Fruity, sweet	1471	1471	58.43 ± 9.99	0.19 ^b^	308
22	Benzaldehyde	100-52-7	Bitter almond-like, green	1530	1522	870.21 ± 29.32	0.35 ^b^	2486
23	3,7-Dimethyl-6-octen-3-ol	18,479-51-1	Floral, sweet	1512	1531	28.07 ± 9.43	300 ^c^	<1
24	Linalool	78-70-6	Floral, sweet, woody	1550	1546	1331.53 ± 150.53	0.87 ^a^	1530
25	1-Octanol	111-87-5	Citrus-like, green, sweet	1539	1555	54.45 ± 9.66	0.13 ^b^	419
26	Terpinen-4-ol	562-74-3	Earthy, moldy	1581	1597	112.34 ± 11.26	0.59 ^b^	190
27	Menthol	1490-04-6	Mint-like, fresh	1635	1637	176.66 ± 12.54	0.1 ^b^	1767
28	*γ*-Caprolactone	695-06-7	Coconut-like, fruity, sweet	1678	1684	21.31 ± 5.56	16 ^b^	1
29	Terpineol	98-55-5	Pine, terpene, green	1700	1688	1106.38 ± 135.93	0.15 ^b^	7376
30	Citral	5392-40-5	Green, citrus, sweet, woody, fresh	1716	1720	3.87 ± 0.82	0.12 ^b^	32
31	Citronellol	1117-61-9	Soapy, rose-like	1765	1762	75.24 ± 6.56	0.04 ^b^	1881
32	cis-3,7-Dimethyl-2,6-octadien-1-ol	106-25-2	Rose-like, flowery	1806	1762	55.2 ± 25.9	0.68 ^b^	81
33	*β*-Damascenone	23,726-93-4	Sweet, floral, fruity	1806	1807	147.84 ± 18.16	0.0009 ^d^	164,266
34	Geraniol	106-24-1	Rose-like, sweet, floral	1830	1842	159.8 ± 15.32	1.1 ^a^	145
35	Benzyl alcohol	100-51-6	Honey, sweet, floral, fruity	1844	1863	36.14 ± 5.94	0.62 ^b^	58
36	*γ*-Octanoic lactone	104-50-7	Coconut-like, sweet	1898	1894	94.96 ± 4.09	0.024 ^a^	3957
37	*β*-Ionone	79-77-6	Floral, sweet, violet	1923	1925	274.7 ± 10.42	0.007 ^b^	39,243
38	(*E*)-Methyl cinnamate	1754-62-7	Sweet, fruity	2080	2054	28.41 ± 2.43	0.67 ^c^	42
39	Dihydrojasmone lactone	7011-83-8	Jasmine, sweet, floral	-	2104	4.25 ± 0.84	-	-
40	Ethyl cinnamate	103-36-6	Sweet, floral, wine, fruity	2104	2109	141.92 ± 11.12	0.17 ^b^	835
41	*γ*-Decalactone	706-14-9	Peach-like, coconut-like	2126	2122	1458.77 ± 103.8	1.1 ^a^	1326
42	Nonanoic acid	112-05-0	Moldy, pungent	2144	2153	24.43 ± 2.38	3000 ^c^	<1
43	*γ*-Undecalactone	104-67-6	Peach-like, sweet	2259	2262	660.65 ± 75.32	2.1 ^a^	315
44	*γ*-Dodecalactone	2305-5-7	Coconut-like, sweet	2340	2345	84.22 ± 5.52	0.43 ^a^	196
45	Benzyl benzoate	120-51-4	Floral, sweet, face cream like	2636	2640	97.52 ± 7.36	0.341 ^b^	286

^a^, threshold values of the aroma compounds referenced from the reference [12,22]; ^b^, referenced from the book [23]; ^c^, referenced from the reference [24]; ^d^, detected threshold value in the water matrix.

## Data Availability

The original contributions presented in the study are included in the article/Supplementary Material, further inquiries can be directed to the corresponding author.

## Supplementary Materials

The following supporting information can be downloaded at: https://www.mdpi.com/article/10.3390/foods13081266/s1. Figure S1: The dominance of sensations results of eleven kinds of sport drink (A, B, C, and D represented the pineapple, peach, orange, and lime flavor of MI, respectively; E, represented the classic flavor of HI-classic flavor; F, Fruity flavor of HA; G, grapefruit flavor of SC; H, Blueberry flavor of GA; I, J, and K represented the Blueberry, Lemon, and Orange flavor of VI, respectively); Figure S2. TIC chromatogram of MI-peach.
