# Supplementary material for: Decoding the Effect of Running on Flavor Perception Changes during Consumption of Sports Drinks
Source: Foods. 2024 Apr 20;13(8):1266. doi: 10.3390/foods13081266 (PMC11049042; doi:10.3390/foods13081266)
Supplement: Supplementary file 1 [file foods-13-01266-s001.zip › foods-2964089-supplementary.pdf]

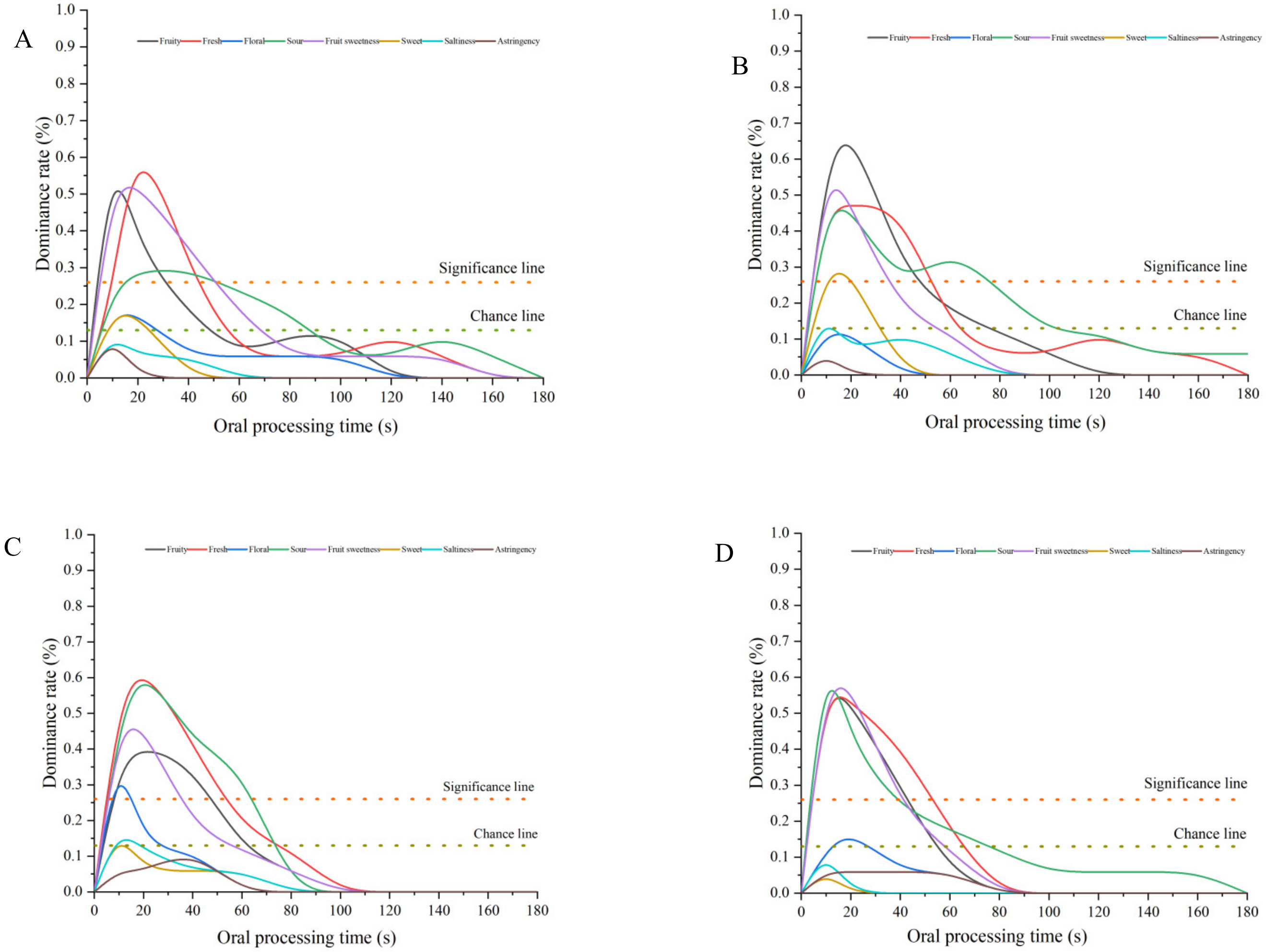
A
B
C
D
Fruity
Fresh
Floral
Sour
Fruit sweetness
Sweet
Saltiness
Astringency
Significance line
Chance line
Dominance rate (%)
Oral processing time (s)
0.0
0.1
0.2
0.3
0.4
0.5
0.6
0.7
0.8
0.9
1.0
0
20
40
60
80
100
120
140
160
180

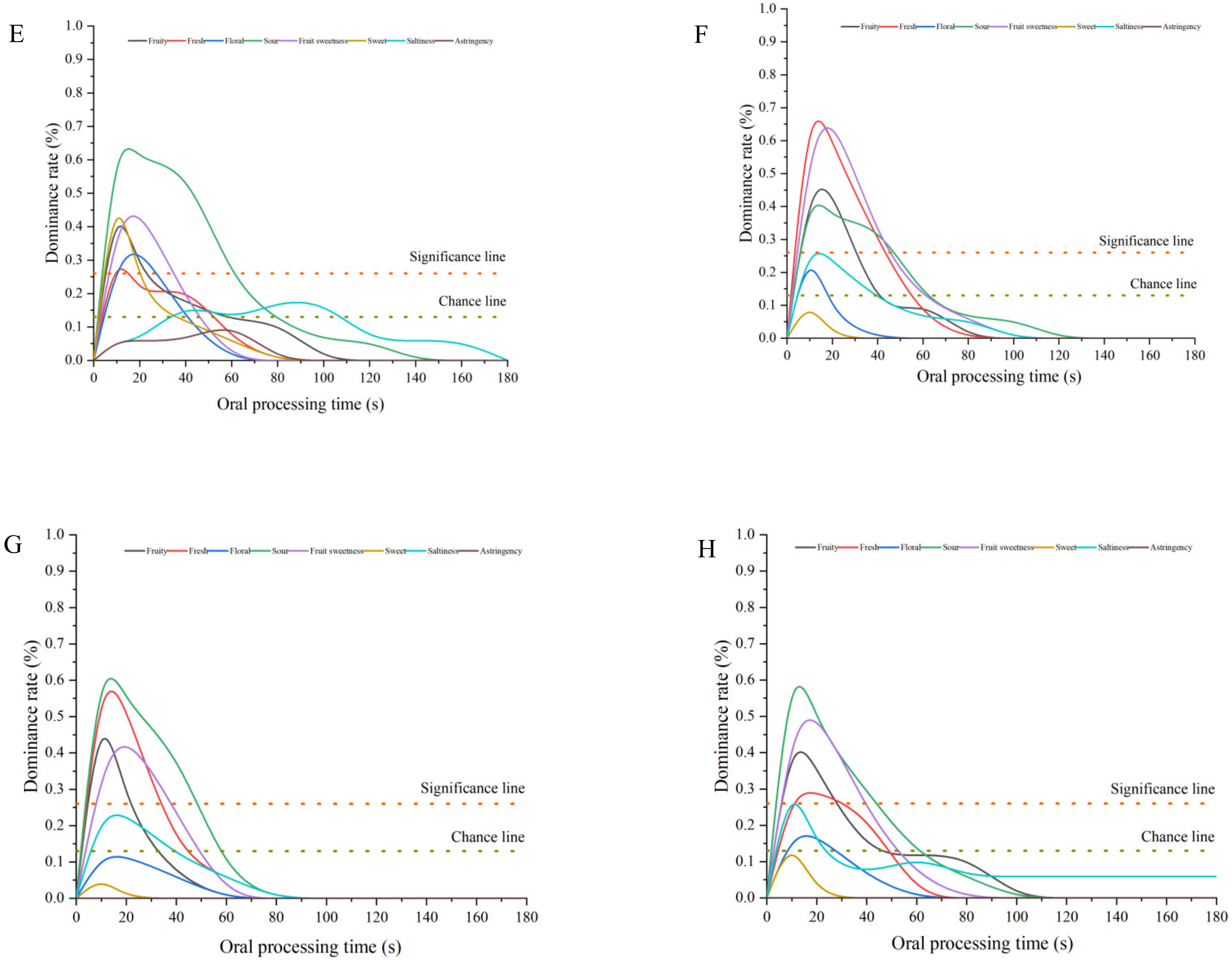
E
F
G
H
Fruity
Fresh
Floral
Sour
Fruit sweetness
Sweet
Saltiness
Astringency
Significance line
Chance line
Dominance rate (%)
Oral processing time (s)

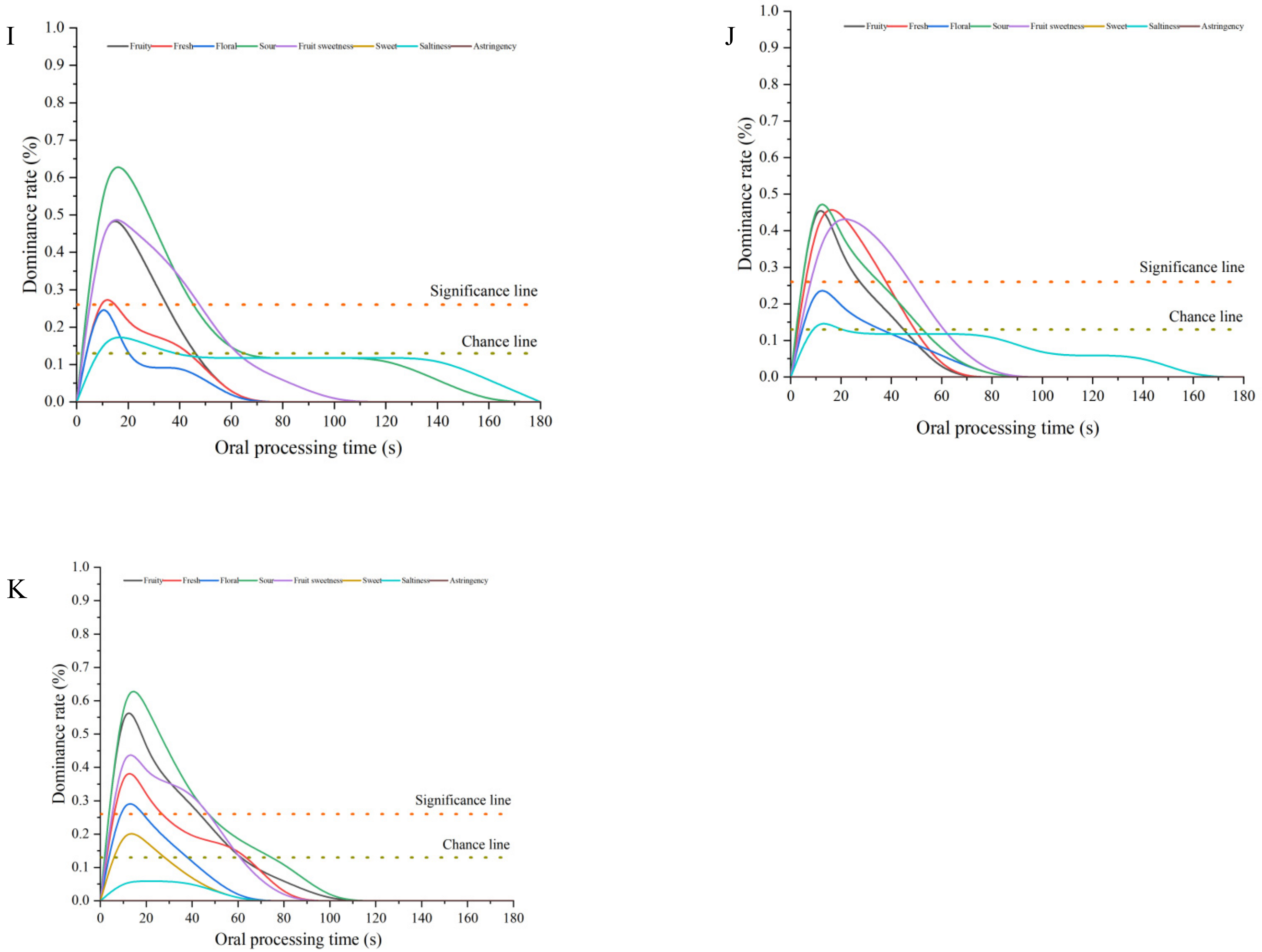

I
J
K
Fruity
Fresh
Floral
Sour
Fruit sweetness
Sweet
Saltiness
Astringency
Significance line
Chance line
Dominance rate (%)
Oral processing time (s)
0.0
0.1
0.2
0.3
0.4
0.5
0.6
0.7
0.8
0.9
1.0
0
20
40
60
80
100
120
140
160
180

Figure S1. The temporal dominance of sensations results of eleven kinds of sport drink (A, B, C, and D represented the pineapple, peach, orange, and lime flavor of MI, respectively; E, represented the classic flavor of HI-classic flavor; F, Fruity flavor of HA; G, grapefruit flavor of SC; H, Blueberry flavor of GA; I, J, and K represented the Blueberry, Lemon, and Orange flavor of VI, respectively).

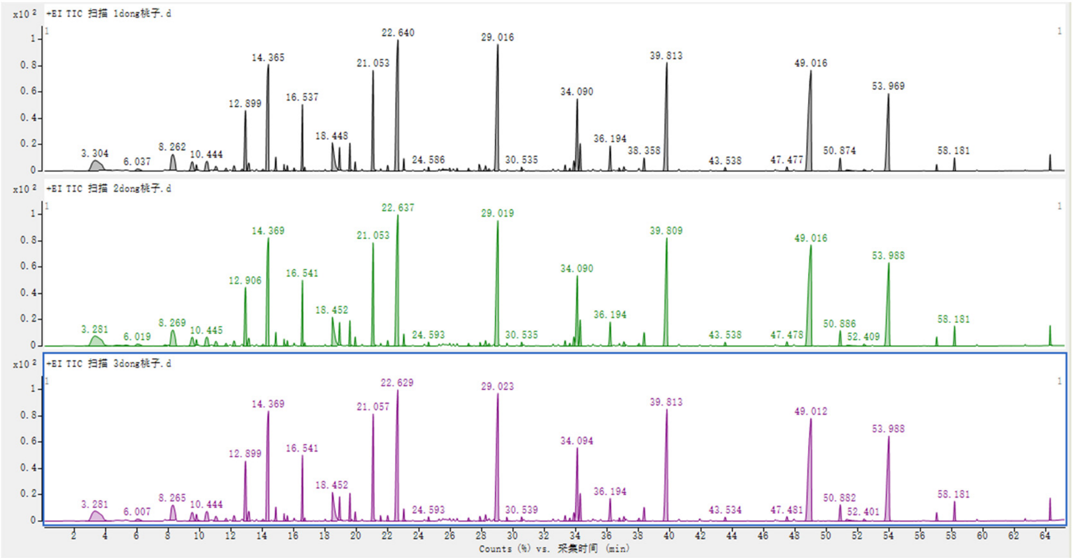

Figure S2. TIC chromatogram of MI-peach
